# Correction to: Fibroblast growth factor receptor 3 alterations and response to immune checkpoint inhibition in metastatic urothelial cancer: a real world experience

**DOI:** 10.1038/s41416-022-01781-y

**Published:** 2022-03-11

**Authors:** Tracy L. Rose, William H. Weir, Gregory M. Mayhew, Yoichiro Shibata, Patrick Eulitt, Joshua M. Uronis, Mi Zhou, Matthew Nielsen, Angela B. Smith, Michael Woods, Michele C. Hayward, Ashley H. Salazar, Matthew I. Milowsky, Sara E. Wobker, Katrina McGinty, Michael V. Millburn, Joel R. Eisner, William Y. Kim

**Affiliations:** 1 Lineberger Comprehensive Cancer Center, University of North Carolina at Chapel Hill, Chapel Hill, NC USA; 2 Department of Medicine, University of North Carolina at Chapel Hill, Chapel Hill, NC USA; 3 GeneCentric Therapeutics, Inc., Durham, NC USA; 4 Department of Urology, University of North Carolina at Chapel Hill, Chapel Hill, NC USA; 5 Department of Urology, Loyola University, Chicago, IL USA; 6 Department of Pathology and Laboratory Medicine, University of North Carolina at Chapel Hill, Chapel Hill, NC USA; 7 Department of Radiology, University of North Carolina at Chapel Hill, Chapel Hill, NC USA; 8 Department of Genetics, University of North Carolina at Chapel Hill, Chapel Hill, NC USA

**Keywords:** Bladder cancer, Cancer immunotherapy

Correction to: *British Journal of Cancer* 10.1038/s41416-021-01488-6, published online 22 July 2021

The original version of this article unfortunately contained an error. The labels were swapped in Figure 3a. The WT should be purple and the FGFR altered should be red. The original article has been corrected.

